# Supplementary material for: Abscisic Acid Mediates Salicylic Acid Induced Chilling Tolerance of Grafted Cucumber by Activating H2O2 Biosynthesis and Accumulation
Source: Int J Mol Sci. 2022 Dec 16;23(24):16057. doi: 10.3390/ijms232416057 (PMC9783703; doi:10.3390/ijms232416057)
Supplement: Supplementary file 1 [file ijms-23-16057-s001.zip › Table S1.pdf]

Table S1 The primer sequences(5'→3') of real-time qPCR

Gene	Primer sequences
CsActin	F: AGAAGATCTGGCATCACA<br>R: TCCAATCCAGACACTGTACT
CmActin	F: CACGAAACTACCTACAACTCC<br>R: CTCATCCTGTCAGCAATAC
CsCBF1	F: TACAGAGGAGTCAGGAGGA<br>R: AGAATCGGCGAAATTGA
CsCOR	F: TGTTCAAGAGGGTGGTGTCG<br>R: GGATCGGGTGAGTTTCTCCA
PAL	F: ATGGCTTCATATTGCTCTGAG<br>R: ATGCCTCAAGTCAATTGCTTG
NCED	F: TGGTGAACCGAAATCTACTTG<br>R: CGAAGGCTAAGATGTGGC
RBOH1	F: AAGGTTGCTGTTTATCC<br>R: AATGGTCTTGAGTTGGG
