# Supplementary figures and images for: Abscisic Acid Mediates Salicylic Acid Induced Chilling Tolerance of Grafted Cucumber by Activating H2O2 Biosynthesis and Accumulation
Source: Int J Mol Sci. 2022 Dec 16;23(24):16057. doi: 10.3390/ijms232416057 (PMC9783703; doi:10.3390/ijms232416057)

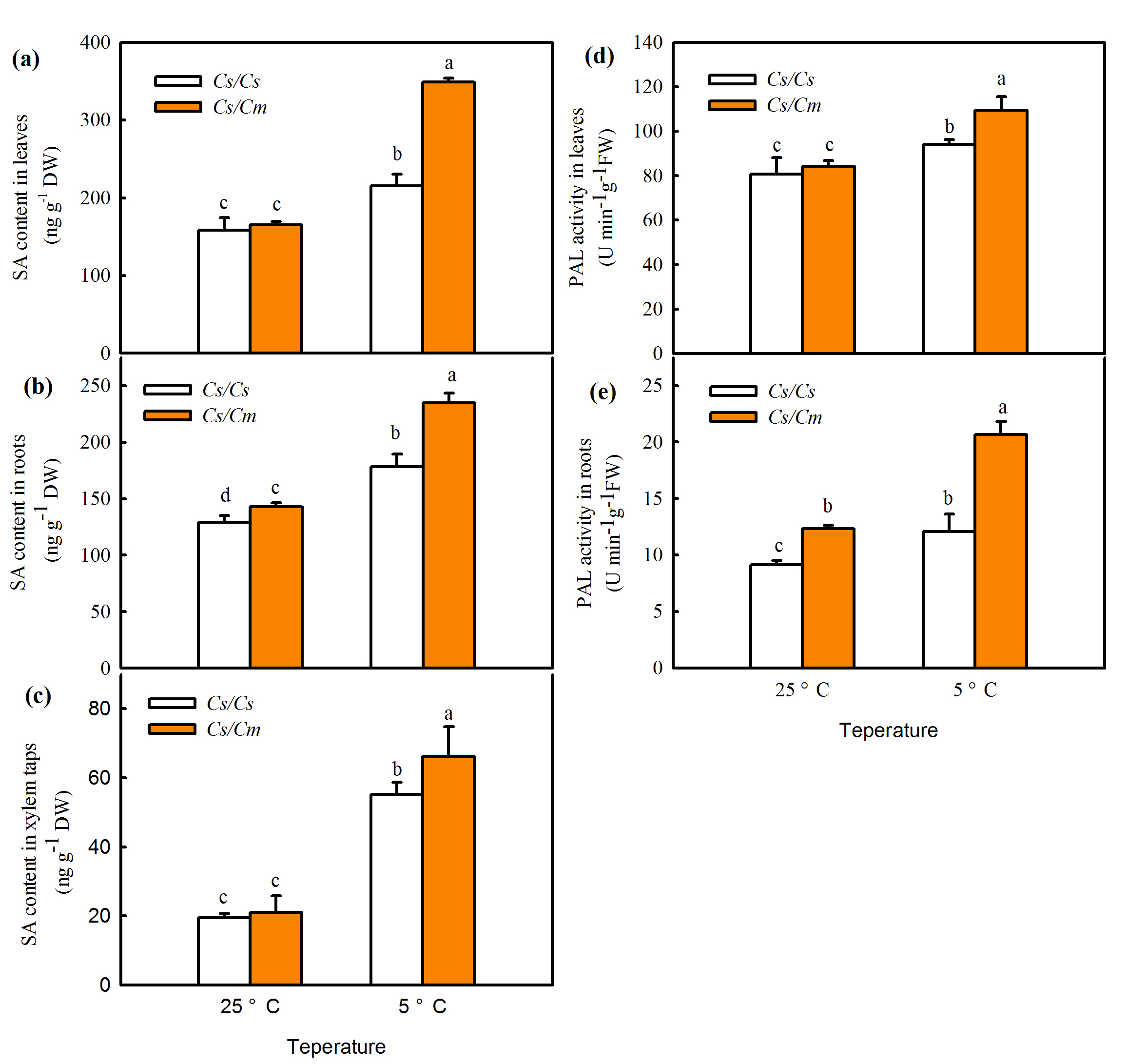

Supplement: Supplementary file 1 [file ijms-23-16057-s001.zip › Figure S1.JPG]

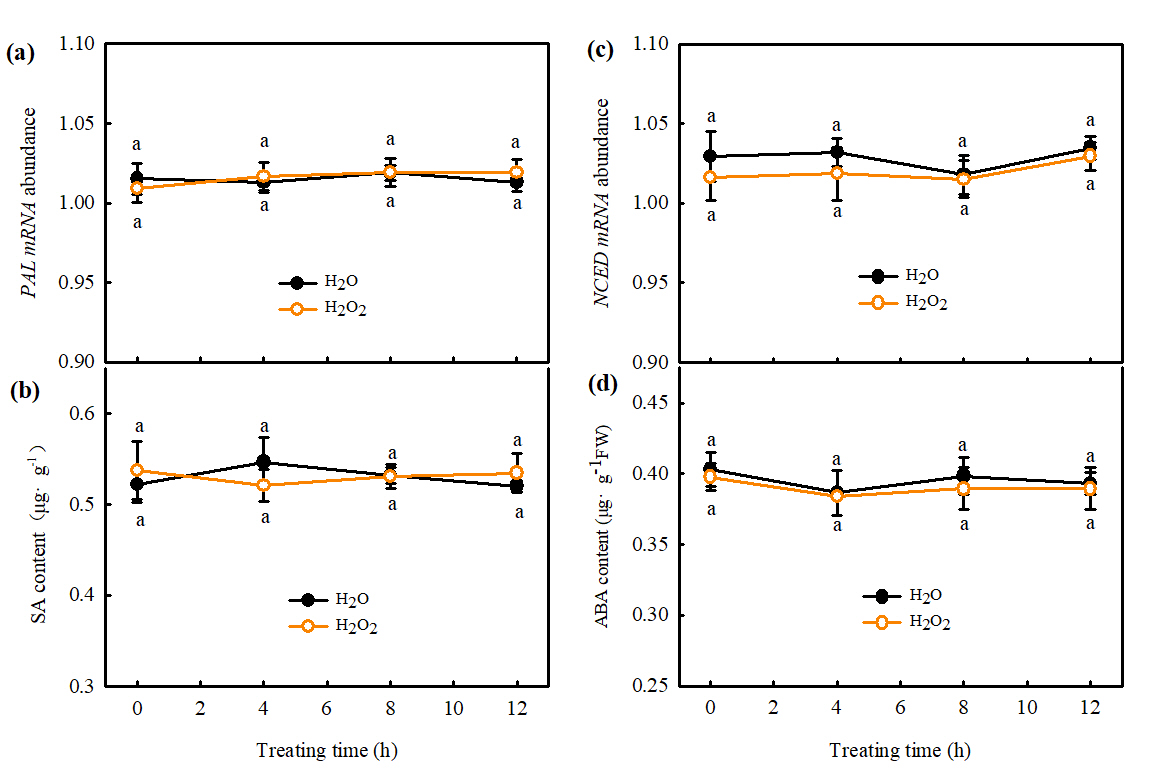

Supplement: Supplementary file 1 [file ijms-23-16057-s001.zip › Figure S2.JPG]
